# Perinatal outcomes in twin pregnancies complicated by maternal morbidity: evidence from the WHO Multicountry Survey on Maternal and Newborn Health

**DOI:** 10.1186/s12884-018-2082-9

**Published:** 2018-11-20

**Authors:** Danielly S. Santana, Carla Silveira, Maria L. Costa, Renato T. Souza, Fernanda G. Surita, João P. Souza, Syeda Batool Mazhar, Kapila Jayaratne, Zahida Qureshi, Maria H. Sousa, Joshua P. Vogel, José G. Cecatti, on behalf of the WHO Multi-Country Survey on Maternal and Newborn Health Research Network

**Affiliations:** 1 Department of Obstetrics and Gynecology, University of Campinas, Alexander Fleming Street, 101, Campinas, SP 13083-891 Brazil; 2 UNDP/UNFPA/UNICEF/WHO/World Bank Special Programme of Research, Development and Research Training in Human Reproduction (HRP), Department of Reproductive Health and Research, World Health Organization, Geneva, Switzerland; 3 Pakistan Institute of Medical Sciences, Islamabad, Pakistan; 4 Maternal & Child Morbidity & Mortality Surveillance Unit, Family Health Bureau, Ministry of Health, Colombo, Sri Lanka; 5 Department of Obstetrics and Gynecology, University of Nairobi, Nairobi, Kenya; 6 Department of Public Health, Jundiai Medical School, Jundiai, Brazil

**Keywords:** Twin pregnancy, Perinatal outcome, Maternal morbidity

## Abstract

**Background:**

Twin pregnancy was associated with significantly higher rates of adverse neonatal and perinatal outcomes, especially for the second twin. In addition, the maternal complications (potentially life-threatening conditions-PLTC, maternal near miss-MNM, and maternal mortality-MM) are directly related to twin pregnancy and independently associated with adverse perinatal outcome. The objective of the preset study is to evaluate perinatal outcomes associated with twin pregnancies, stratified by severe maternal morbidity and order of birth.

**Methods:**

Secondary analysis of the WHO Multicountry Survey on Maternal and Newborn Health (WHOMCS), a cross-sectional study implemented in 29 countries. Data from 8568 twin deliveries were compared with 308,127 singleton deliveries. The occurrence of adverse perinatal outcomes and maternal complications were assessed. Factors independently associated with adverse perinatal outcomes were reported with adjusted PR (Prevalence Ratio) and 95%CI.

**Results:**

The occurrence of severe maternal morbidity and maternal death was significantly higher among twin compared to singleton pregnancies in all regions. Twin deliveries were associated with higher rates of preterm delivery (37.1%), Apgar scores less than 7 at 5th minute (7.8 and 10.1% respectively for first and second twins), low birth weight (53.2% for the first and 61.1% for the second twin), stillbirth (3.6% for the first and 5.7% for the second twin), early neonatal death (3.5% for the first and 5.2% for the second twin), admission to NICU (23.6% for the first and 29.3% for the second twin) and any adverse perinatal outcomes (67% for the first twin and 72.3% for the second). Outcomes were consistently worse for the second twin across all outcomes. Poisson multiple regression analysis identified several factors independently associated with an adverse perinatal outcome, including both maternal complications and twin pregnancy.

**Conclusion:**

Twin pregnancy is significantly associated with severe maternal morbidity and with worse perinatal outcomes, especially for the second twin.

## Background

Every year more than 10 million infants die before their fifth birthday and 8 million even before their first year of life. Over 6.3 million perinatal death occurred worldwide, in the year 2000, most of them in developing countries [1]. Global efforts and strategies have been aimed at reducing these numbers, including the fourth Millennium Development Goal and the new Sustainable Development Goals, which include ending preventable deaths of newborns and children under 5 years of age in its third goal [2, 3]. However, it is important to understand the magnitude of perinatal and neonatal morbidity and mortality to address their determinants [1].

Among the obstetric conditions known to increase the risk of perinatal mortality, twin pregnancy is a well-recognized factor [4–6] Twin pregnancy results from a complex interaction of genetic and environmental determinants (maternal age, parity, family history of multiple pregnancies, habits, social conditions) occurring in approximately 2–4% of livebirths and interestingly, rates are highest in some parts of Africa where care is often poorest [7–10]. However, its incidence increased more than 70% globally in the last three decades mainly in high and middle-income countries due to the use of assisted reproductive technologies [8, 9, 11, 12]. Twin pregnancy is associated with a number of obstetric complications, some of them with serious perinatal consequences, especially for the second twin [10, 13]. The rate of perinatal mortality can be up to six times higher in twin compared to singleton pregnancies, largely due to higher rates of preterm delivery and fetal growth restriction seen in twin pregnancies [4, 5, 10]. Preterm birth and birth weight are also significant determinants of morbidity and mortality into infancy and childhood [5].

The risk of maternal mortality is approximately 2.5 times higher in twin than in singleton pregnancies [8]. Maternal death (MD) is understood as the last stage of a continuum of increasingly severe morbidity, which may occur in pregnancy and is preceded by any potentially life-threatening conditions (PLTC) and by maternal near miss (MNM) [14]. Research has been interested in the relationship of twin pregnancies and severe maternal morbidity. A secondary analysis was recently conducted using data from the WHO Global Survey on Maternal and Perinatal Health (2004–2008), where twin pregnancy was a significant, independent risk factor for maternal and perinatal morbidity and mortality compared to singleton pregnancies [6]. A more recent secondary analysis from the WHO Multicountry Survey on Maternal and Newborn Health (WHOMCS, 2010–2011) explored the association of twin pregnancy with adverse maternal outcomes using the MNM criteria, reporting a 3 times higher risk of MNM and a 4 times higher risk of MD among twin pregnancy than in singleton [15]. These analyses, however, did not explore or report on any associations with adverse perinatal outcomes.

The current study aims to asses in the WHOMCS database the prevalence of potentially life-threatening conditions, maternal near miss and maternal death between twin and singleton pregnancies by regions. Then, considering the birth order, to evaluate the prevalence of perinatal outcomes (preterm births, Apgar Score at 5th min < 7, fetal death, neonatal death, perinatal death, neonatal intensive care unit admission, adequacy of weight for gestational age) between singleton versus twin. In addition, it aims to identify sociodemographic, obstetric characteristics and the occurrence of maternal complications in singleton and twin deliveries associated with any adverse perinatal outcome.

## Methods

The WHOMCS was a cross-sectional study performed to assess the maternal and perinatal morbidity and mortality in 359 institutions from 29 countries (Afghanistan, Angola, Argentina, Brazil, Cambodia, China, Democratic Republic of the Congo, Ecuador, India, Japan, Jordan, Kenya, Lebanon, Mexico, Mongolia, Nepal, Nicaragua, Niger, Nigeria, Pakistan, Palestine, Paraguay, Peru, Philippines, Qatar, Sri Lanka, Thailand, Uganda, Vietnam), from May 2010 to December 2011. This is a secondary analysis of the database from this worldwide network. Methodological details of the WHOMCS have been previously published elsewhere [16, 17].

Briefly, the survey was conducted in a network of health facilities in Latin America, Africa, Asia and the Middle East, the same that had previously participated in the WHO Global Survey on Maternal and Perinatal Health (2004–2008) [18]. Countries, provinces, and health facilities were randomly selected through a stratified multistage cluster sampling strategy. Countries in each region were selected with a probability proportional to population size. In each country, three sub-regions were also selected: the capital plus two other randomly selected provinces. In each province, seven health facilities with at least 1000 deliveries annually and full capacity for performing caesarean sections were randomly selected. Data was collected from two to four months depending on the annual number of deliveries in each institution. The coordination of the study was of World Health Organization in Geneva; each region had a regional coordinator; each country had a country coordinator; each province had province coordinator, and each facility had a local coordinator who was responsible for selecting some health professional staff to collect data.

Trained data collectors identified eligible subjects in participating facilities. Eligible participants were all women who gave birth during the data collection period in the participating facilities with their respective newborns and all women who were admitted with a severe maternal outcome (maternal death or maternal near miss) up to seven postpartum days, independently of gestational age and delivery status. Data were collected from the time of admission to death, discharge or 7 days postpartum/post-abortion (whichever came first), irrespective of gestational age and type of delivery. Adverse outcomes occurring after discharge or during a subsequent readmission were not reported.

A paper form was developed with the following variables, maternal and newborns individual data, data related to pregnancy outcomes, severe complications and their management and characteristics of each health facility. This paper form was reviewed by other researchers and pre-tested on a convenient sample of records and clinical settings; the final version was translated to the main language of each participating country. The medical records were reviewed and the data was completed in the paper form, after that, it was entered into a web-based data management system developed for this purpose; the regional data managers monitored the data flow and the quality of data using data validation and progress reports automatically generated by the system. All instructions regarding eligibility criteria, identification, sociodemographic and obstetric characteristics, maternal complications, neonatal complications, and characteristics of deliveries were standardized in a manual of operations used for training and study operationalization. The training also included workshops at country and facility level and a pilot phase to test the complete data management process.

The study protocol was approved by the WHO Ethical Review Committee and by relevant Institutional Review Boards in participating countries and institutions. The WHOMCS was a study of anonymized data, extracted from medical records (with no contact with women) and therefore individual consent was not required.

**Fig. 1:**
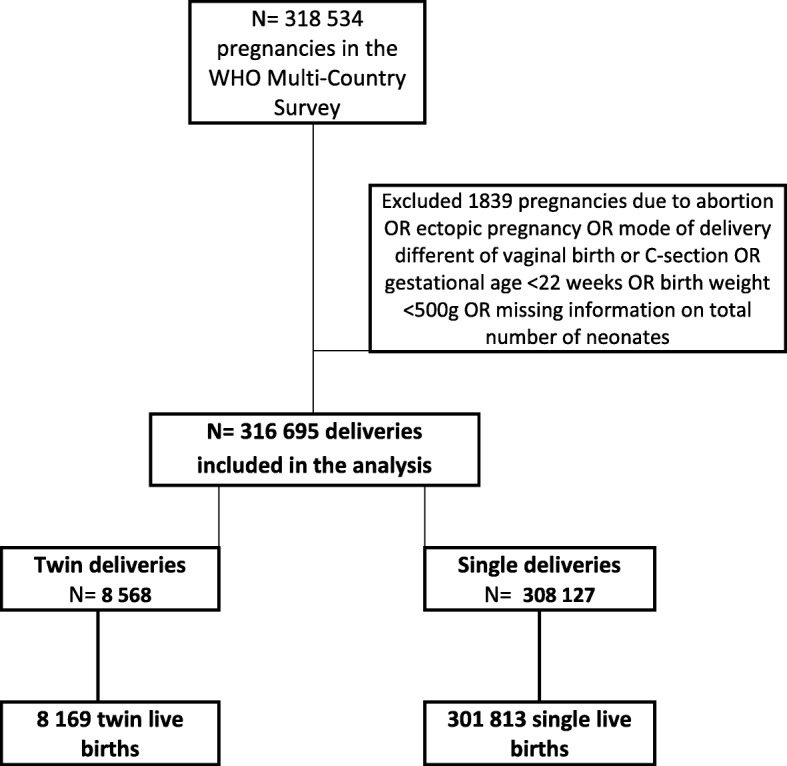
Flowchart of women in the analysis for adverse perinatal outcomes associated with twin pregnancy (each twin = one delivery)

At the end of the data collection, 316,695 deliveries were included with complete information on pregnancy outcomes [17]. For this analysis, twin deliveries were compared with singleton deliveries. To define the study groups, 1839 pregnancies with the following conditions were excluded: pregnancies resulting in abortion or ectopic pregnancy; neonate weighing less than 500 g or with no information on birthweight; less than 22 weeks of gestation; and missing data on termination of pregnancy, final mode of delivery or abortion, and total number of neonates delivered. Analyses were based on 8568 twins and 308,127 singletons (Fig. 1).

### Statistical analysis

**Fig. 2:**
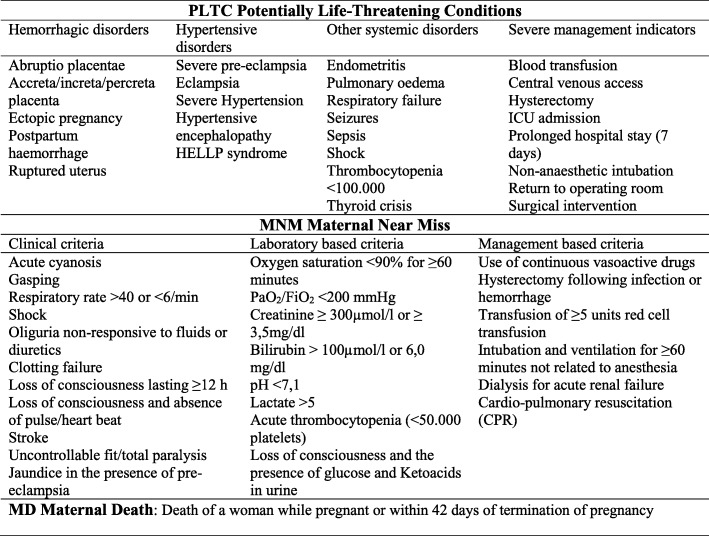
Definitions of severe maternal complications according to the World Health Organization [14]. Portions reprinted from Say L, Souza JP, Pattinson RC; WHO working group on Maternal Mortality and Morbidity classifications. Maternal near miss—towards a standard tool for monitoring quality of maternal health care. Best Pract Res Clin Obstet Gynaecol 2009; 23:287–96, with permission from Elsevier. *HELLP* hemolysis, elevated liver enzymes, low platelet count, *ICU* intensive care unit, *CPR* cardiopulmonary resuscitation

The occurrence of maternal outcomes (potentially life-threatening conditions, maternal near miss, maternal death and no complications, according to the WHO definitions and criteria [14]– Fig. 2) was assessed by continent for twin and singleton pregnancies. For this step, women were the unit of analysis. Statistical significance of differences between twins and singletons was assessed by χ^2^ tests. The diagnostic criteria used to characterize women with potentially life-threatening conditions, maternal near miss and maternal death are those recommended by WHO (Fig. 2) [14, 17].

For assessing perinatal outcomes, the unit of analysis was neonates (regardless of vital status at birth). Each newborn corresponds to one delivery, so pregnancy resulting in two newborns is considered as two deliveries. We used several perinatal outcomes: Apgar score less than 7 at 5 min, fetal death (the death of a fetus from 22 completed weeks or 500 g until before birth), early neonatal death (intra-hospital neonatal death in first week of life, occurring prior to discharge), late fetal death (the death of a fetus from 28 weeks until before birth), perinatal death (early neonatal death plus fetal death), preterm birth (birth before 37 weeks gestation), neonatal intensive care unit admission (NICU), and small-for-gestational-age (defined as the weight at childbirth below the 10th percentile for the correspondent gestational age). In addition, we developed two composite outcomes– acute adverse perinatal outcome (AcAPO: Apgar score less than 7 at 5 min, or perinatal death, or neonatal intensive care unit admission) and any adverse perinatal outcome (APO: Apgar score less than 7 at 5 min, or perinatal death, or neonatal intensive care unit admission, or small-for-gestational-age). All perinatal outcomes were separately reported for the first and the second twins, using Prevalence Ratios adjusted by the cluster design effect (PR_adj_). Comparisons were performed in three steps to assess if they differed by birth order: first twins versus singletons; second twins versus singletons; and second versus first twins. The adequacy of weight for gestational age in the present analysis was evaluated based on Fenton growth chart [19]. The Fenton growth chart is based on the growth target recommended for preterm infants, has specific graphics for girls and boys, and the chart is designed to allow tracing how children are measured, this growth chart was chosen due to the high prevalence of preterm birth in the present study [19].

Differences in sociodemographic, obstetric characteristics and maternal complications (PLTC, MNM, and MD) among twins or singletons according to the occurrence of any adverse perinatal outcome were estimated using χ^2^ test.

Finally, a Poisson multiple regression analysis was performed to identify factors independently associated with adverse perinatal outcomes. For that, a regression model was built using acute adverse perinatal outcome and any adverse perinatal outcome as the main outcomes and all other variables as predictors, including the information on the pregnancy being twin or singleton, and the occurrence of maternal complications. The resultant Prevalence Ratios were therefore adjusted not only for the survey design but also for all other predictors (PR_adj_). Results were considered significant when the estimated *p*-values were below 0.05. All statistical procedures were performed using SPSS (Version 20.0) and Stata (Release 7) programs. Results were reported in accordance with the STROBE statement [20].

## Results

Among the 318,534 women initially enrolled in the WHOMCS, 312,867 women and 316,695 deliveries remained after the exclusion criteria were applied, 4756 (1.5%) of them with twin pregnancies corresponding to 8568 deliveries of neonates (Fig. 1). Table 1 shows the occurrence of potentially life-threatening conditions, maternal near miss and maternal death by region comparatively between twin and singleton pregnancies. All regions showed significantly higher occurrence of maternal complications and maternal death for twin pregnancies. Rates were consistently higher for the African and Asian regions than for Latin America.

**Table 1:** PLTC, MNM and MD for twin and singleton pregnancies by region. WHO Multicountry Survey, 2010–2011

Region	Twin pregnancies				Singleton pregnancies				*p*- value
	NC (%)	PLTC (%)	MNM (%)	MD (%)	NC (%)	PLTC (%)	MNM (%)	MD (%)	
Africa	1219 (84·2)	196 (13·5)	27 (1·9)	5 (0·3)	67,547 (93·3)	4181 (5·8)	495 (0·7)	139 (0·2)	**< 0·001**
Asia	2078 (85·8)	299 (12·4)	32 (1·3)	12 (0·5)	161,118 (94·2)	9129 (5·3)	686 (0·4)	131 (0·1)	**< 0·001**
Latin America	735 (82·8)	141 (15·9)	11 (1·2)	1 (0·1)	58,412 (90·3)	5935 (9·2)	314 (0·5)	24 (< 0·1)	**< 0·001**
TOTAL	4032 (84·8)	636 (13·4)	70 (1·5)	18 (0·4)	287,077 (93·2)	19,245 (6·2)	1495 (0·5)	294 (0·1)	**< 0·001**

*MD* maternal death, *MNM* maternal near miss, *NC* No complication, *PLTC* potentially life-threatening condition

*P* value referring to the comparison between no complication/any complication in twin vs singleton

**Fig. 3:**
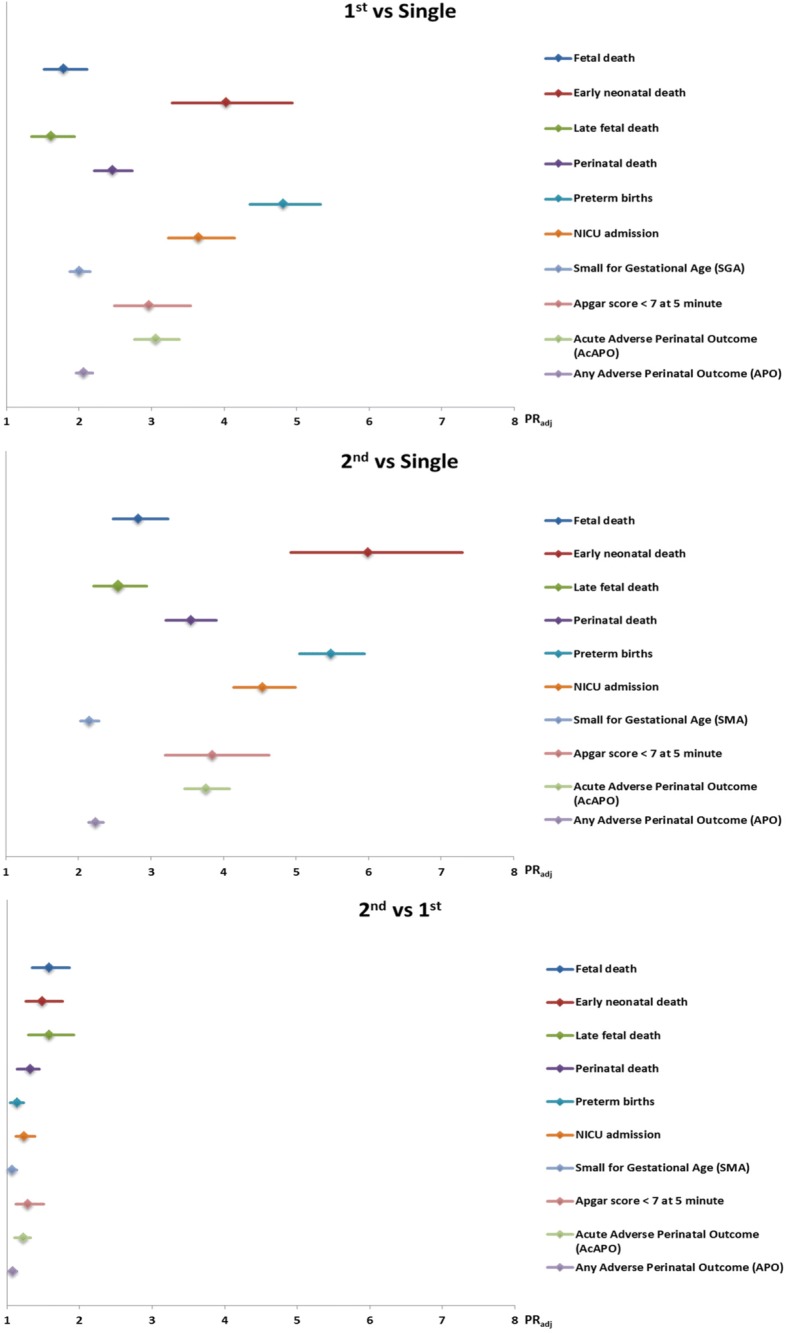
Estimated risks (PR_adj_, 95% CI) of some adverse perinatal outcomes for the first and second twins comparatively with singletons, and second comparatively with first twins

Table 2 shows that twin deliveries were associated with higher rates of preterm birth (< 37 weeks), early preterm birth (< 34 weeks), low birth weight, small for gestational age, and Apgar score less than 7. For all perinatal outcomes, rates were significantly higher for twins compared to singletons, and also for the second twin compared to the first twin (Fig. 3).

**Table 2:** Perinatal outcomes in twin and singleton deliveries (unity of analysis are neonates). WHO Multicountry Survey, 2010–2011

Perinatal outcomes	Twin deliveries n (%)		Singleton deliveries n (%)	Total n (%)	PR_adj_ (95% CI)		
Gestational age at delivery ^a^							
< 34 weeks	1098 (13·0)		7337 (2·4)	8435 (2·7)	**6·77 (5·99–7·66)**		
34–36 weeks	2035 (24·1)		14,791 (4·9)	16,826 (5·4)	**5·57 (5·03–6·17)**		
≥ 37 weeks	5312 (62·9)		282,801 (92·7)	288,113 (91·9)	Ref.		
Birth weight ^b^	**1st twin**	**2nd twin**			**1st vs Single**	**2nd vs Single**	**2nd vs 1st**
< 2500 g	2495 (53·2)	2299 (61·1)	32,480 (10·6)	37,274 (11·8)	**5·03 (4·59–5·52)**	**5·78 (5·41–6·17)**	**1·15 (1·06–1·24)**
≥ 2500 g	2193 (46·8)	1464 (38·9)	274,665 (89·4)	278,322 (88·2)	Ref.	Ref.	Ref.
Adequacy of weight for GA ^c^							
**SGA**	2385 (51.7)	2043 (55.2)	77,855 (25.7)	82,283 (26.4)	**2.01 (1.88–2.15)**	**2.15 (2.03–2.28)**	**1.07 (1.01–1.13)**
**No SGA**	2232 (48.3)	1658 (44.8)	225,285 (74.3)	229,175 (73.6)	Ref.	Ref.	Ref.
Apgar Score at 5th min ^d^							
< 7	352 (7·8)	364 (10·1)	7928 (2·6)	8644 (2·8)	**2·97 (2·49–3·54)**	**3·85 (3·20–4·63)**	**1·29 (1·12–1·50)**
7–10	4142 (92·2)	3227 (89·9)	292,805 (97·4)	300,174 (97·2)	Ref.	Ref.	Ref.
Fetal and neonatal outcomes							
Fetal death ^e^	169 (3·6)	215 (5·7)	6151 (2·0)	6535 (2·1)	**1·79 (1·52–2·11)**	**2·83 (2·48–3·23)**	**1·58 (1·35–1·86)**
Early neonatal death ^f^	160 (3·5)	189 (5·2)	2636 (0·9)	2985 (1·0)	**4·03 (3·29–4·94)**	**5·99 (4·93–7·29)**	**1·49 (1·26–1·76)**
Late fetal death ^g^	130 (2·7)	165 (4·3)	5241 (1·7)	5536 (1·7)	**1·62 (1·35–1·94)**	**2·55 (2·21–2·94)**	**1·58 (1·30–1·92)**
Perinatal death ^h^	328 (7.0)	381 (10.0)	8706 (2.8)	9415 (3.0)	**2.46 (2.21–2.73)**	**3.55 (3.21–3.90)**	**1.44 (1.14–1.32)**
Preterm births ^a^	1634 (35·0)	1492 (39·8)	22,128 (7·3)	25,254 (8·1)	**4·82 (4·36–5·33)**	**5·48 (5·05–5·94)**	**1·14 (1·05–1·23)**
NICU admission ^i^	1073 (23·6)	1059 (29·3)	19,468 (6·4)	21,600 (7·0)	**3·65 (3·23–4·14)**	**4·54 (4·14–4·99)**	**1·24 (1·12–1·38)**
Acute Adverse Perinatal Outcome (AcAPO) ^j^	1395 (29.9)	1390 (36.8)	30,006 (9.8)	32,791 (10.4)	**3.06 (2.77–3.38)**	**3.76 (3.47–4.08)**	**1.23 (1.11–1.32)**
Any Adverse Perinatal Outcome (APO) ^k^	3101 (67.0)	2709 (72.3)	98,128 (32.4)	103,938 (33.3)	**2.07 (1.96–2.19)**	**2.24 (2.14–2.34)**	**1.08 (1.03–1.13)**
Total	**4733**	**3811** ^**l**^	**308,127**	**316,671**			

Chi-square test adjusted for the cluster design effect

Missing information for a: 3320; b: 1075; c: 5213; d: 7853; e: 178; f: 6761; g: 159; h: 375; i: 6638; j: 1508; k: 4712 neonates

Values in bold mean they are statistically significant (*p* < 0.05)

*AcAPO* Acute Adverse Perinatal Outcome (Apgar score at 5th min < 7 OR Perinatal death OR NICU admission)

*APO* any Adverse Perinatal Outcome (Apgar score at 5th min < 7 OR Perinatal death OR NICU admission OR SGA)

^l^There is no available information for the second or higher twin for the countries Paraguay, Peru, Philippines, Qatar, Thailand, Vietnam and Uganda

The occurrence of any adverse perinatal outcome (APO) was more frequent among twin deliveries in women between 18 and 35 years (87,1%), with lower maternal education (55,3% with 0–9 years of education), higher parity, with a partner, in preterm birth, whose delivery was through an elective C-section. The gestational age at delivery for singleton pregnancies was 37 weeks or more in about 90% of cases, while in twin pregnancies this prevalence was approximately 65%. In addition, all maternal morbid conditions were more frequent among twins than singletons and more associated with any APO (Table 3).

**Table 3:** Sociodemographic and obstetric conditions among twin and single deliveries according to the occurrence of any Adverse Perinatal Outcome (APO). WHO Multicountry Survey, 2010–2011

Perinatal outcomes	Twin deliveries n (%)		Singleton deliveries n (%)		Total	*p*-value*
	APO	No APO	APO	No APO		
Maternal age^a^						< 0.001
< 18	49 (1.6)	22 (1.4)	3987 (4.1)	6971 (3.4)	11,029	
18–35	2693 (87.1)	1296 (85.0)	86,375 (88.3)	179,196 (87.6)	269,560	
> 35	349 (11.3)	206 (13.5)	7510 (7.7)	18,420 (9.0)	26,485	
Maternal education (years)^b^						< 0.001
0–4	694 (24.6)	259 (18.1)	21,295 (23.4)	34,647 (18.3)	56,895	
5–9	865 (30.7)	414 (28.9)	29,664 (32.6)	57,190 (30.3)	88,133	
> 10	1263 (44.8)	758 (53.0)	40,025 (44.0)	97,219 (51.4)	139,265	
Parity^c^						< 0.001
0	1121 (36.3)	401 (26.3)	47,293 (48.3)	81,773 (39.9)	130,588	
1–2	1306 (42.3)	793 (52.1)	37,017 (37.8)	88,631 (43.3)	127,747	
> 2	661 (21.4)	329 (21.6)	13,681 (14.0)	34,487 (16.8)	49,158	
Marital status^d^						0.013
With no partner	251 (8.2)	91 (6.0)	10,898 (11.2)	19,766 (9.7)	31,006	
With partner	2825 (91.8)	1422 (94.0)	86,434 (88.8)	183,915 (90.3)	274,596	
Gestational age at delivery^e^						< 0.001
< 34 weeks	488 (15.8)	74 (4.8)	5966 (6.1)	1329 (0.6)	7857	
34–36 weeks	619 (20.1)	435 (28.5)	5850 (6.0)	8838 (4.3)	15,742	
≥ 37 weeks	1972 (64.0)	1017 (66.6)	86,010 (87.9)	195,007 (95.0)	284,006	
Onset of labour^f^						< 0.001
Spontaneous	3902 (67.2)	1611 (62.9)	74,825 (76.4)	159,268 (77.7)	239,606	
Induced	455 (7.8)	162 (6.3)	11,572 (11.8)	20,638 (10.1)	32,827	
Elective C-section	1446 (24.9)	787 (30.7)	11,561 (11.8)	25,054 (12.2)	38,848	
Mode of delivery^g^						< 0.001
Vaginal birth	1575 (50.8)	691 (45.3)	70,422 (71.8)	146,340 (71.3)	219,028	
Cesarean section	1525 (49.2)	835 (54.7)	27,701 (28.2)	58,834 (28.7)	88,895	
Maternal complications^h^						< 0.001
No complications	2606 (84.1)	1312 (86.0)	88,416 (90.1)	194,250 (94.7)	286,584	
PLTC	425 (13.7)	200 (13.1)	8567 (8.7)	10,365 (5.1)	19,557	
MNM	53 (1.7)	13 (0.9)	921 (0.9)	507 (0.2)	1494	
MD	16 (0.5)	1 (0.1)	221 (0.2)	52 (0.0)	290	
Total	5810	2562	98,128	205,178	311,678	
	8372		303,306			

*design-based *p*-value

Missing information for: a: 9597; b: 32378; c: 9178; d: 11069; e: 9066; f: 5390; g: 8748; and h: 8746 cases

*APO* any Adverse Perinatal Outcome (Apgar score at 5th min < 7 OR Perinatal death OR NICU admission OR SGA)

*MD* maternal death, *MNM* maternal near miss, *PLTC* potentially life-threatening condition

The factors independently identified as protective for acute or any adverse perinatal outcome were the higher gestational age at birth, vaginal delivery, parity ≥1 and maternal age (Table 4). Any maternal complications (PLTC, MNM and MD) and twin pregnancy were both identified as the main factors associated with a higher risk of APO.

**Table 4:** Factors independently associated with Acute Adverse Perinatal Outcome (AcAPO) and with any Adverse Perinatal Outcome (APO) by Poisson multiple regression analysis. WHO Multicountry Survey, 2010–2011

Model/ Variables	PR_adj_	95% CI	p
AcAPO [*n* = 283,549]			
Gestational age at delivery (weeks)	0.81	0.80–0.82	< 0.001
Maternal complications (PLTC, MNM, MD)	1.88	1.76–2.02	< 0.001
Group (Twin)	1.50	1.40–1.61	< 0.001
Mode of delivery (Vaginal)	0.64	0.58–0.70	< 0.001
Parity (≥1)	0.86	0.81–0.91	< 0.001
Marital status (With no partner)	1.43	1.12–1.83	0.004
Maternal education (Up to nine years)	1.18	1.04–1.33	0.011
Maternal age (years)	1.006	1.001–1.011	0.014
**APO** [*n* = 283,393]			
Group (Twin)	1.87	1.78–1.97	< 0.001
Maternal complications (PLTC, SMM, MD)	1.39	1.32–1.46	< 0.001
Parity (≥1)	0.82	0.80–0.85	< 0.001
Gestational age at delivery (weeks)	0.96	0.95–0.97	< 0.001
Maternal education (Up to nine years)	1.23	1.15–1.32	< 0.001
Maternal age (years)	0.99	0.986–0.994	< 0.001

*AcAPO* Acute Adverse Perinatal Outcome (Apgar score at 5th min < 7 OR Perinatal death OR NICU admission)

*APO* any Adverse Perinatal Outcome (Apgar score at 5th min < 7 OR Perinatal death OR NICU admission OR SGA)

PR_adj_ = prevalence ratio adjusted for cluster effect; 95% CI: 95% confidence interval for prevalence ratio

Poisson multiple regression analysis, controlled by: Group (Single:0; Twin: 1); Age (years); Maternal education (Up to nine years: 1; > 10 years: 0); Parity (0/≥1: 1); Marital status (With no partner: 1/With partner: 0); Gestational age at delivery (weeks); Onset of labor (Spontaneous: 0; Other: 1); Mode of delivery (Vaginal: 1; C-section: 0); Maternal complications (No complication: 0/PLTC, MNM, MD: 1)

## Discussion

Twin pregnancy has increased risks of preterm labor, spontaneous preterm birth, premature rupture of membranes, neonatal and perinatal morbidity and mortality [5, 10, 21, 22]. The occurrence of any potentially life-threatening conditions, maternal near miss or maternal death was twice as high or more, in twin pregnancies; they had complications in 15.3% while singleton pregnancies had only in 6.8%. Results were reasonably consistent across geographical regions. These outcomes were the object of study in at least another two articles with data from World Health Organization detailing the relationship between twin pregnancy and severe maternal morbidity [6, 15]. No explanations were found to variable rates of adverse maternal outcomes in twin pregnancies in different countries with similar income, however it may relate to differences in the quality of available care and local complication patterns [6, 15].

The reported preterm birth rates among twins are very similar to that found in other studies, ranging from 31% [6, 22] to 44% [23], but some reporting up to 63% [24]. Early preterm births are less frequent than late (34–36 weeks), as Vogel et al. reported in the WHO Global Survey, with 11.9% of preterm birth below 34 completed weeks [6]. Higher early preterm rates are important, as they are associated with higher neonatal morbidity and perinatal death rates, mainly due to respiratory complications [6, 23, 25, 26].

Low birth weight is also more frequent among twin pregnancies. A previous study found that this risk was 8.3 times higher than in singletons, with a mean birth weight of 2300 g [24], higher than that observed in our study (5 times higher). This risk is associated with the increase in Apgar score at 5th minute < 7 and death during the first year of life [22–24, 27]. Adequacy of weight for gestational age better assesses the size of the fetus for a given gestational age (compared to birth weight alone). This is particularly useful in populations where preterm birth rates are high. A fetus that is small for gestational age is more likely to experience perinatal morbidity and mortality and adverse effects in adult life [28]. Few studies have evaluated this outcome among twin deliveries, but associations between twin pregnancies and higher rates of small-for-gestational-age have been reported [28, 29]. For these estimations, we used the curves of Fenton et al. [19] because we believed that it was more appropriate to be used when the prevalence of preterm birth is very high, as is the case among twin pregnancies in this population. However, due to the number of cases to have such estimates, it was not feasible to have such assessment performed using different nomograms for comparison.

The risk for low 5th minute Apgar score was three times higher for twin pregnancy (either for the first or second twin) than for singletons. Additionally, it was 1.3 times higher for the second when both twins were compared. This significantly lower Apgar score for the second twin is always taken into consideration in discussions about the best mode of delivery for twin pregnancies and the time interval between first and second twin, although not justifying an indication for a systematic Cesarean section for twin pregnancies [6, 30–32]. The higher rates of admission to a neonatal intensive care unit we found have also been reported by previous studies on the topic [6, 31].

Prevalence of fetal death of one of the twins varies from 0,5-6,8% with the worst result for monochorionic pregnancy presenting a high prevalence for this condition (50–70%) and risk for the surviving fetus including the fetal death of this co-twin, neurological morbidity and iatrogenic preterm delivery [33, 34]. In the current study, we have not data on chorionicity, however fetal death (death after 28 weeks) occurred over 1.5 times (3.6%) for the first twin and almost 3 times (5.7%) for the second twin when compared to singletons (2.0%).

Perinatal death has been described as up to four times higher in twin pregnancies than in singletons, mainly due to preterm birth, fetal growth restriction, low Apgar scores and extremely low birth weight [5, 6, 23, 25]. In our study, it was found to be 2.5 times higher for the first twin and 3.5 for the second one. This difference between both twins has already been described [31]. In the current study, we also observed a higher risk for fetal and early neonatal death, supporting previous findings from other studies [6, 31].

Cesarean section, including that performed electively, was the most common mode of delivery in twin pregnancy in the present study. The debate on the best mode of delivery is extensive, especially considering higher adverse outcomes for the second twin, and that neither labor nor vaginal delivery is associated with worse perinatal outcomes, since the first twin is in cephalic presentation. There is currently no indication for a policy of planned cesarean delivery, although still some controversies frequently arise among professionals [11, 12, 30, 35, 36].

In the current analysis higher rates of maternal complications are directly related to twin pregnancy (15.9% in twins with APO, 14.1% in twins with no APO and 9.8% in singletons with APO and 5.3% in singletons with no APO). This reinforces some recent studies identifying twin pregnancy as a risk factor for the occurrence of severe maternal morbidity. In a WHO Global Survey analysis, Vogel et al. reported a 1.85 higher risk of occurrence of a severe maternal outcome (maternal death, admission to an intensive care unit, blood transfusion or hysterectomy) between twin pregnancies compared to singletons [6]. Using the new WHO diagnostic criteria for severe maternal conditions, another recent study from our group using the same database identified that twin pregnancies increased twofold the risk of occurrence of PLTC, threefold the risk of MNM and fourfold of occurrence of MD compared with singleton pregnancies [15]. These differences reinforce that twin pregnancy is associated with worse outcomes for both newborns and women. Whether this justifies the need for a more specialized care for women with a twin pregnancy, not only aiming at a good perinatal outcome but also for the maternal outcome, is not completely understood and deserves more specific studies [5, 6, 12, 15, 37].

In the multivariate analysis, both twin pregnancy and maternal complications (PLTC, MNM and MD) still appear as factors independently associated with acute or any adverse perinatal outcome. As already argued, a twin pregnancy is associated with a number of perinatal complications either acute or chronic. However, the relationship between adverse perinatal outcomes and severe maternal conditions reinforces that when the woman develops an adverse condition, the fetus suffers direct consequences (growth restriction and stillbirth) or indirectly by the need of interrupting pregnancy before term, with all the consequences of being preterm. In view of these results and knowing that twin pregnancy is not a modified condition but maternal complications are preventable conditions through improvement of the quality of obstetric care theattention to the pregnant woman is able to modify the perinatal outcomes associated with a twin pregnancy.

There were a few limitations to our study. We had no data on the chorionicity and pregnancies archived by ART/FIV for twin pregnancies – what could be associated with perinatal outcomes. In addition, there is no information at all on ethnicity and BMI of the women, what could also be associated with both twin pregnancy and perinatal outcomes. This was a big international multicenter study that for collecting information on all deliveries during a period of time should use a very short questionnaire to facilitate data collection. Considering twin pregnancy was not the main objective of the study, these variables were not included. Despite all quality control procedures some inconsistency could occur unnoticed from data collected from each individual women with a paper form until the feeding of the electronic database, either by the reviewer or by the system. The reported patterns probably relate more to Low- and Middle-Income Countries settings as a result of the included countries, and therefore the generalization of results for High-Income countries may be limited. In addition, this WHOMCS was mainly performed in secondary and tertiary facilities functioning as referral hospitals with a probable over-representation of maternal complications and maternal/perinatal deaths and considering that the results are based on the facility, countries with low health-facility coverage will be underrepresented, in those facilities the results will probably be lower mainly to severe maternal morbidity. These data might not be representative of maternal outcomes and coverage of essential interventions in smaller facilities or in the community, with variations among countries. In addition, the data collection included only women and newborns up to seven days postpartum or abortion with that cases progressed to maternal and neonatal complications beyond this period may be lost.

On the other hand, we could also highlight some strengths of the current study. The WHOMCS is a large, multi-country database and based on information collected in a standardized way; outcome data on more than 8000 twins were captured, and results obtained identify worse perinatal outcomes for twins, especially for the second and the association between severe maternal morbidity and twin pregnancy. These findings allow the understanding that twin pregnancy is not only associated with obstetric complications but also maternal death. In the clinical practice, these results could assist in the implementation of protocols for identification of risk conditions and maternal and perinatal care.

## Conclusion

In this analysis, twin pregnancy was associated with significantly higher rates of adverse neonatal and perinatal outcomes. Our results confirm previous observations of increased perinatal mortality and morbidity; outcomes for the second twin were generally poorer than for the first twin. Despite the limitations discussed above, for being a multicenter study, ours finding confirm the necessary identification of higher risk cases and referral to high complexity facilities with capacity for quality prenatal care and intensive care for newborns. Further studies on the topic would be welcome in the future, especially to assess whether specialized obstetric and neonatal care is able to reduce the occurrence of some complications, thus improving maternal and perinatal outcomes.

## Abbreviations

AcAPO: Acute adverse perinatal outcome
APO: Any adverse perinatal outcome
MD: Maternal death
MM: Maternal mortality
MNM: Maternal near miss
NICU: Neonatal intensive care unit
PLTC: Potentially life-threatening conditions
PR: Prevalence Ratio
WHO: World Health Organization
